# Trumpism, climate and COVID: Social bases of the new science rejection

**DOI:** 10.1371/journal.pone.0293059

**Published:** 2024-01-10

**Authors:** Lawrence C. Hamilton

**Affiliations:** Department of Sociology, University of New Hampshire, Durham, New Hampshire, United States of America; Carleton University Faculty of Public Affairs, CANADA

## Abstract

Although the hazards posed by greenhouse warming and COVID-19 are quite different, diagnosis and mitigation prospects for both depend heavily on science. Unfortunately, the reality of both threats has been subject to politicized science rejection in the US, making these deadly problems less tractable. There are substantial parallels between the two cases of science rejection, including common rhetoric and conservative political leadership. Survey research has reached widely-replicated conclusions regarding the social bases of climate-change perceptions. Corresponding studies of COVID-19 perceptions have found some political commonalities, but less agreement on other details. Here, we address this gap using generalized structural equation modeling (GSEM) and 2021 US survey data to make direct comparisons between the social bases of rejecting the reality of anthropogenic climate change (ACC) and rejecting COVID-19 vaccination. Trumpism, operationalized from approval of ex-president Trump, is viewed as an intervening variable that influences both types of science rejection. Trumpism itself is predicted by age, race, evangelical religion, ideology, and receptivity to seemingly non-political conspiracy beliefs. Considering direct as well as indirect effects (through Trumpism), climate change and vaccine rejection are similarly predicted by white and evangelical identity, conspiracism, and by education×ideology and friends×party interactions. The finding that Trumpism exacerbates science rejection could also apply to other science- and expertise-related topics unrelated to climate and COVID. These results invite broader comparisons across topics, with analogous movements in other countries, and continued tracking as US Trumpism evolves beyond Trump.

## 1. Introduction

Climate change has long been recognized by scientists as a major problem—potentially an existential threat to social and ecological systems [1,2]. The SARS-Cov2 virus with its variants emerged more recently but quickly attained major threat status as well, causing over one million known deaths in the US and six million worldwide in its first two years; the true totals are likely much higher [3,4]. Although the hazards posed by greenhouse warming and COVID-19 are quite different, diagnosis and mitigation prospects for both depend heavily on science. Unfortunately, scientific analysis, policy discussions, and even the basic reality of both threats have become targets of politicized science rejection in the US, making these deadly problems less tractable.

The arguments and tactics deployed against scientists and science-based mitigation for climate change on one hand, and COVID-19 on the other, have some basic elements in common [5–7]. Science rejection in each case has been led by conservative media and political leaders, encouraging the wide partisan divisions seen on general-public surveys. Although these commonalities are clear, they leave further hypotheses worth testing. In what other respects do the social bases of science rejection on COVID-19 resemble those of climate change, which have been established through more than 40 years of research on environmental concern [8,9]?

While general correlations between political identity and views on climate change or COVID-19 are well known, we have less knowledge regarding common (or divergent) patterns with other individual characteristics including age, gender, education, religion, race or income. The more immediate personal impacts of COVID-19 compared with climate change, and its direct links to individual behavior, might give rise to differences in demographic patterns. Certain interaction effects relating to information uptake that have been noted with climate change and environmental issues also remain untested regarding COVID-19. Finally, there are new elements in the US political landscape—notably the mainstreaming of conspiracy beliefs and the authoritarian, grievance-focused emergence of Trumpism.

The analysis that follows tests Trumpism and openness to conspiracy beliefs alongside more familiar demographic characteristics as predictors of rejecting the reality of human-caused climate change and COVID-19 vaccination. Data are from a nationally representative US survey conducted in mid-2021, when Trump was no longer in office. At that time vaccinations had been approved and made widely available, but employer mandates were not yet in effect. Because Trumpism emerged recently, growing out of traditional conservatism but discarding many of its tenets, it is modeled here as an intervening variable that might be predicted by some of the same demographic characteristics as climate and COVID responses, while contributing further direct effects of its own.

## 2. Background

Conservative political identity has long been associated with lower concern about environmental problems [9–11], including the reality of anthropogenic climate change or ACC [8,12,13], and climate mitigation policies [14,15]. Politics also played a large role in shaping the evolution of US policy on COVID-19, affecting public perceptions of its seriousness as well as support for public-health countermeasures from social distancing to mask wearing and vaccination. General-public surveys find strong associations between political identity and individual COVID-related behavior [16–21], while aggregate analyses show corresponding associations between county or state-level political indicators and public-health outcomes such as stay-at-home compliance, vaccination and death rates [22–26].

Survey researchers historically operationalized political identity in terms of ideology or party, two indicators that have become more overlapping in the US due to polarization and party sorting [27–30]. Another political indicator used in some recent studies has been support for president/ex-president Trump. When tested, Trumpism (operationalized as Trump voting or approval) exhibits additional effects beyond ideology or party, but comparable to them in strength [14,31–33]. While drawing from a traditional conservative base, Trumpism incorporates stronger elements of personality cult, authoritarianism and scapegoating, with lower or even reversed emphasis on traditionally conservative values such as personal morality, anticommunism and limited government. Shao and Hao [19] present arguments and empirical support for treating Trump support as an intervening variable; a similar approach is taken in this paper.

Conspiratorial thinking has been a conspicuous feature of US politics at least since at least the 1950s [34] and appears to be growing in prominence across a wide range of topics [35,36]. Although some conspiracy beliefs focus on political leaders (e.g., Obama is Muslim, or Trump really won the 2020 election), many others have been directed against scientists [37], on topics including climate change [38,39], vaccinations [39] and COVID-19 [5,40–42]. There are conspiracy beliefs discounting both anthropogenic climate change and the seriousness of COVID-19. Showing that specifically climate or COVID-related beliefs predict other views on those same topics seems circular, however. The analysis that follows instead tests an indicator for receptivity to extreme conspiratorial beliefs that have *not* been much politicized and are unrelated to climate or COVID.

Multiple studies on the social bases of views on climate change and other environmental problems have replicated two interactions involving political identity. The most common are information×political-identity effects in which, for example, concerns about climate change increase with education among liberals and moderates, but do not increase and may even decrease with education among the most conservative. Variations include party, ideology or worldview used as political-identity indicators; and education [12,14,43–47], self-assessed understanding [13,48], tested science literacy [49,50], or numerical literacy [51,52] as information indicators. Similar interactions have been found with non-climate dependent variables relating to vaccines [53], perceptions of local flooding or weather trends [54–56], renewable energy [57], and other environment-related issues [58,59]. In all these findings the interactions take a similar form, often graphed with a right-facing megaphone shape: the best-educated partisans stand the farthest apart (e.g., Fig 3A–3D in [12]).

Such information×identity-type interactions reflect the influence of partisan cueing—absorbing cues from partisan elites, co-partisans, or (negatively) perceived antagonists, regarding which beliefs are appropriate for your identity—as a heuristic in “forming” your own views on difficult issues [60–64]. Better-educated or otherwise information-rich individuals tend to be more sensitive to partisan cues. The public-opinion impacts of messaging from political and partisan-media elites have been prominent regarding both climate change [27,61,64–67] and COVID-19 [18,68,69]. Information-processing behaviors such as *biased assimilation* [13,70] and *motivated reasoning* [71–75], to the extent these are stronger among information elites, likely contribute to the observed information×identity interactions as well.

A second, less dramatic interaction effect follows from survey reports that Americans increasingly choose to associate with people of their own political persuasion, a pattern that intensifies polarization [76–78]. Political identity×friends-type interactions have been confirmed in several recent studies involving local weather trends [56], wildlife management [79], and local weather or climate [80]. Their common form is that political-identity effects are stronger, or partisan gradients steeper, when most of the respondent’s friends are reported as belonging to the same political party. This may be especially true among conservatives.

## 3. Research questions and hypotheses

Sociopolitical identity—as indicated by political party, ideology, or most recently support for Donald Trump—has been consistently linked to rejection of both anthropogenic climate change (ACC) and COVID-19 vaccination. It is not established to what extent other basic respondent characteristics such as age, gender, race, income, religion or income also show similar or divergent patterns across these two important cases of science rejection. Exploring such comparisons provides the basic motivation for our analysis. Because the social bases of ACC views have been well studied, we adapt those results into specific hypotheses, tested here using new data on both ACC and COVID-19 vaccine rejection.

H1: Trump support will be greater among older respondents, and those who identify as white, evangelical Christian, conservative and Republican.
H2: Rejecting the reality of ACC, and personal rejection of COVID-19 vaccination, will both be more common among ideologically conservative, Republican and Trump-supporting individuals.
H2a: Support for ex-president Trump thus can be modeled as an intervening (mediating) variable, related to ideology, party and other background characteristics, with both direct and indirect effects on ACC and vaccine rejection.
H3: Individuals who self-identify as white or as evangelical Christians (traits often linked to conservative identity) will be more likely to reject ACC and vaccination.
H4: Education×ideology interaction effects, widely observed in ACC studies, will also affect vaccination responses in similar ways: ideological divisions on both topics widening with education.
H5: Partisan divisions on Trump support, ACC and vaccine rejection will be wider among respondents who report having mostly same-party friends (friends×party interaction).
H6: After controlling for the background, sociopolitical, interaction and identity effects noted above, Trump support, rejection of ACC and rejection of COVID-19 vaccination will also be more common among individuals who are receptive to unrelated conspiracy beliefs.

The next section describes a new survey dataset, and the analytical methods used to test these hypotheses in a unified multivariate model.

## 4. Data and methods

### 4.1 POLES 2021 survey

Data analyzed here are from the Polar, Environment and Science survey, conducted in summer and early fall 2021 (POLES 2021). This representative US survey is well suited to the research questions posed above because it carried both climate and COVID-19 items, along with a range of different indicators for sociopolitical identity and respondent background. The survey also carried a number of conspiracy-belief items [41], several of them on topics that have not been politicized and are unrelated to climate or COVID. Other POLES 2021 questions on science-related topics replicated those asked five years earlier on the POLES 2016 survey [14,81,82]. In addition, the 2021 survey carried new questions reflecting changes in the US political context. Finally, and necessitated by the pandemic-driven shift from telephone interviewing to online data collection, the newer survey carried attention-check items to screen out thoughtless respondents—such as those who answered all the questions too quickly, or “straightlined” their agreement or disagreement with blocks of incompatible statements. Research protocols were approved by the Institutional Review Board for the Protection of Human Subjects in Research at the University of New Hampshire (IRB-FY2021-38), and the online questionnaire distributed in two stages: June/July and September/October 2021. After quality screening, we obtained 1,134 valid completions. Sampling for this survey, organized by Qualtrics, aimed for a nationally representative profile of US adults with respect to age, gender, race, education and political party.

**Table 1** lists variables analyzed here, with response summaries and codes used for modeling. The variables include background characteristics, political identification, and two conspiracy questions, along with three endogenous (intervening or dependent) variables of interest: approval of former president Trump, views on climate change, and COVID-19 vaccination status. Experimentally, we calculated probability weights that made the sample marginally more representative. The weights had little effect on relationships or endogenous variable distributions, however, and caused effect estimates to be less precise (inflated the standard errors). Consequently, weighting is not used in the main analyses that follow. Whether unweighted or weighted, the mean Trump approval (*Trumpism*) was 3.9 on a 1–7 scale; 29 or 30 percent rejected the idea of ACC (*NoACC*); and 22 percent said they were not planning to get vaccinated against COVID-19 (*NoVax*). The fractions rejecting ACC and vaccination appear similar, but these are not the same people: only 9 percent of the sample falls in both camps.

**Table 1:** Variables in this analysis, with response percentages from 2021 survey (n = 1,134) and codes used for modeling (Table 2).

**Endogenous variables**
*Trumpism*—Strongly disapprove of former president Trump (1, 36%); somewhat disapprove (2, 5%); lean toward disapproving (3, 5%); neither approve nor disapprove (4, 7%); lean toward approving (5, 7%); somewhat approve (6, 16%); strongly approve (7, 24%).
*NoACC*—Which of the following do you think is more accurate? Climate change is not happening now (1, 5%); climate change is happening now, caused mainly by natural forces (1, 25%); Climate change is happening now, caused mainly by human activities (0, 63%); don’t know (0, 7%).
*NoVax*—Which of the following describes your own situation, regarding vaccination against COVID-19? I do not plan to be vaccinated (1, 22%); I plan to be vaccinated but have not done so yet (0, 13%); I have received first dose of a 2-shot vaccine (0, 9%); I am fully vaccinated (single dose of 1-shot, or both doses of 2-shot) (0, 56%).
**Exogenous variables**
*Age*—Range 18 to 94 years, mean 48 years.
Gender (*Female*)—Female (1, 51%); Male (0, 47%). Missing values: fewer than 2% identified as non-binary or gave no answer.
Race (*White*)—White non-Hispanic (1, 64%); Black or African American (0, 12%); Hispanic (0, 13%); Asian American (0, 6%); Native American (0, 1%); other or mixed (0, 3%); prefer not to say (0, <1%).
*Income*—Household income less than $15k (1, 12%); $15–30k (2, 16%); $30–70k (3, 36%); $70–120k (4, 20%); $120–200k (5, 11%); over $200k (6, 5%).
*Education*—High school or less (–1, 25%); technical school or some college (0, 25%); college graduate (1, 32%); postgraduate work (2, 17%).
Religion (*Evangelical*)—Identify as Christian Evangelical (1, 19%); other Protestant (0, 12%); other Catholic (0, 22%); other Mormon (0, 1%); Jewish (0, 3%); Muslim (0, 3%); Buddhist (0, 2%); Hindu (0, <1%); atheist (0, 4%); agnostic (0, 4%); something else (0, 14%); nothing in particular (0, 16%).
*Ideology*—Extremely liberal (–3, 11%); fairly liberal (–2, 16%); moderate, lean liberal (–1, 12%); moderate, lean neither (0, 19%); moderate, lean conservative (1, 10%); fairly conservative (2, 16%); extremely conservative (3, 16%).
*Party*—Democrat (–1, 46%); Independent (0, 7%); Republican (1, 42%); other or don’t know (coded as missing, 5%).
*Friends*—Most of my friends prefer the same party I do (1, 46%); most prefer different parties (0, 15%); about evenly divided (0, 25%); don’t know (0, 14%).
*Conspiracist*—Sum of *moonfake*, NASA astronauts did not land on the Moon—disagree (1, 70%), unsure (2, 17%), agree (3, 12%); and *earthflat*, the Earth is flat, not round—disagree (1, 81%), unsure (2, 9%), agree (3, 10%). *Conspiracist* ranges from 2 (disagree with both conspiracies, 64%) to 6 (agree with both conspiracies, 4%).

The questions about flat Earth and Moon landings are unusual for such research, although topic-specific conspiracy beliefs are well known in public discussion of Trump support, climate change and vaccination. In our survey 10 percent agreed the Earth is flat, and 12 percent agreed that NASA did not land on the Moon. A further 9 percent were unsure whether the Earth is flat, and 17 percent unsure about the Moon landings. These results parallel those of other recent nationwide surveys that asked flat Earth or Moon landing questions [83,84]. See [41] for comparisons of flat Earth and Moon landing responses to other science-related statements that are undoubtedly true, such as the Earth is billions of years old, or revolves around the Sun.

Conspiracy responses in the June/July and September/October survey stages were nearly identical. There also were no significant shifts in Trump approval, nor in the proportions rejecting ACC or COVID-19. Vaccination eligibility in the US was rolled out by age group in 2021, so older respondents would have had more time to obtain vaccinations, especially in the survey’s June/July stage. To minimize age bias our coding contrasts those who, at the time of the survey, said they were either fully vaccinated, partly vaccinated, or planning to be vaccinated—versus rejectionists who said they *do not plan* to be vaccinated. The rejectionist fraction showed only a slight, nonsignificant drop from summer to early fall. Because response patterns stayed nearly the same, we pool summer and fall responses for Table 1 and the analyses that follow.

### 4.2 Analytical methods

Among the endogenous variables listed in Table 1, approval of former president Trump (*Trumpism*) ranges from 1 (strongly disapprove) to 7 (strongly approve). Most respondents leaned toward one extreme or the other, leaving few in the middle. An alternative dichotomous coding for this question was considered but proved to work less well, as described in section 5.3. Two other endogenous items, climate change and vaccination, offered non-ordinal response choices. Among these choices, rejecting the scientific consensus on ACC, and rejecting COVID-19 vaccination, have notably adverse consequences—delaying climate-change mitigation efforts, or worsening the pandemic. Both positions have been linked to conservative identity, including Trump support. For analysis here, those responses are represented by the variables *NoACC* (reject anthropogenic climate change) and *NoVax* (reject COVID-19 vaccination).

Among the exogenous variables, *age* is measured in years. *Income*, *education*, *ideology* and *party* are ordinal items that tend to have monotonic and roughly linear effects in large samples. Section 5.3 evaluates alternative specifications using categorical education and party, but finds that these fit less well, so both are treated as approximately continuous for our main analysis. Codes for *education*, *ideology* and *party* have been centered on zero, for use with interaction effects. Indicator variables for race (*white*) and religion (Christian *evangelical*) flag two other factors widely described as correlates of Trump approval and hypothesized to have similar effects on rejection of anthropogenic climate change and COVID-19 vaccination. *Gender* is treated dichotomously here, although our survey question was more nuanced. Ten out of 1,134 respondents identified their gender as nonbinary, and six others declined to answer this question. These subsets are potentially interesting, but too small for interpretable analysis.

Conspiracist ideation has become prominent in US political discourse. The most widespread false conspiracy beliefs relate directly to our variables of interest, such as assertions that Trump really won the 2020 election, climate change is a hoax, or COVID-19 vaccinations contain tracking microchips. Obviously, such conspiracy beliefs correlate with other views on Trump, climate or vaccines. The *conspiracist* variable defined in Table 1 is a less tautological indicator based on receptivity to two extreme beliefs that, unlike Trump, climate or vaccines, have not been explicitly linked to conservative identity: that NASA faked the Moon landings, or that the Earth is flat rather than round. Few respondents in our survey agreed with both views, but more than a third agreed with or were open to at least one.

Trumpism is a comparatively recent and still-evolving phenomenon, unlike party, ideology and other demographic characteristics that have been studied through five decades of “social bases” research [9,85,86]. At Trump rallies in particular, Trumpism manifests as a personality cult flavored by grievances, authoritarianism and white nationalism. In the future, Trump’s political style and the devotion of his base might spread to other leaders. His Republican rivals have been described as trying to “out-Trump Trump” [87,88] or “claim the Trumpist mantle” [89]; retrospective accounts refer to his “historical dominance” and a “Trump era” that did not necessarily end with his presidency. Such descriptions foreshadow Trumpism as a phenomenon persisting beyond Trump. Currently, however, his personality cult appears central, while his targets and positions may shift—as in past reversals regarding COVID scientists and vaccines [18]. It seems best at this point to operationalize Trumpism simply in terms of expressed Trump support, as previous researchers have done, while recognizing that more elaborate content-based measures could be developed and validated in future research.

**Fig 1:**
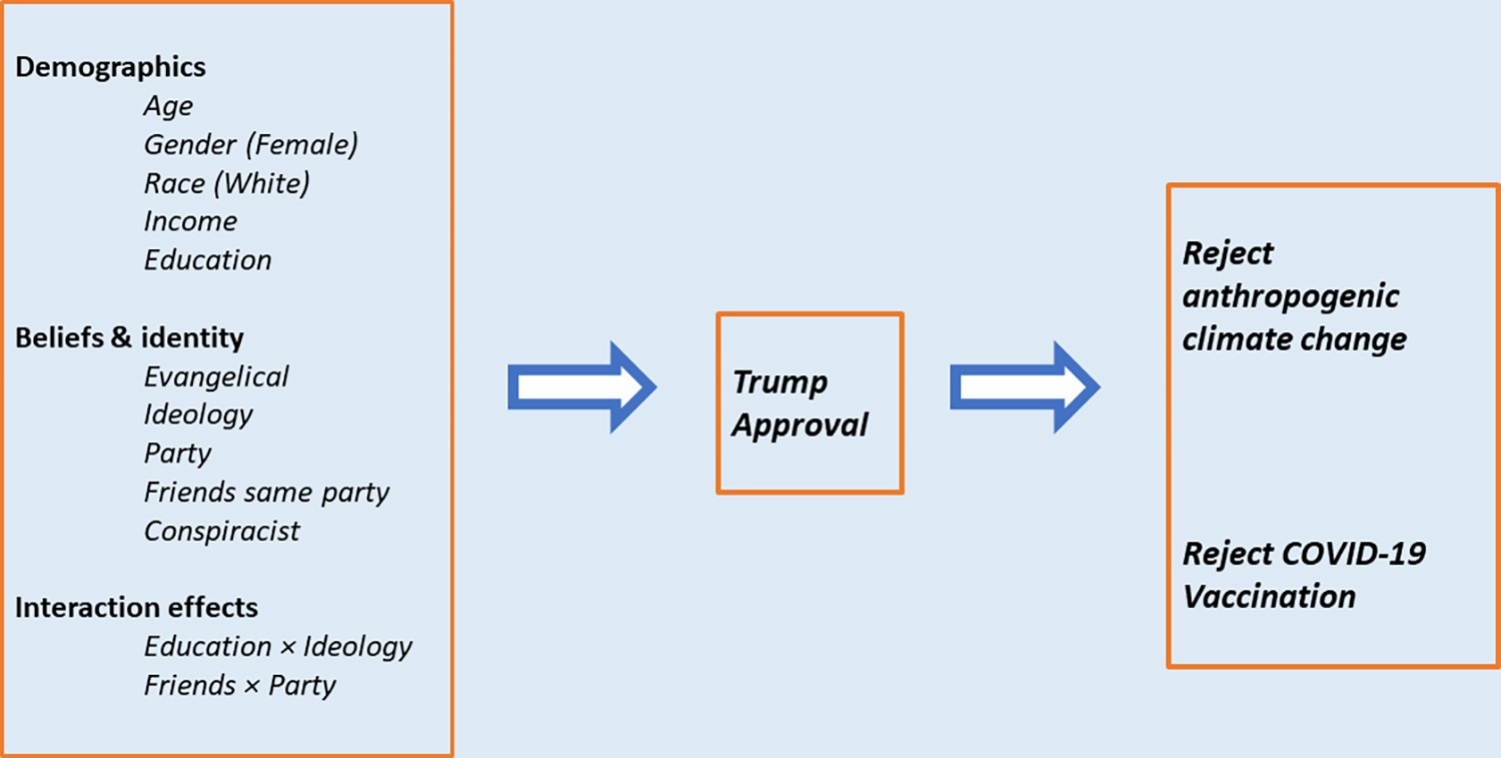
Schematic representation of the generalized structural equation model (GSEM).

As an emergent and currently important sociopolitical identity, Trumpism or Trump support can be depicted as an intervening variable—partly descending from older identity markers (such as ideology and party) and conspiracism, while also contributing direct effects of its own. Intervening-variable hypotheses invoke a structural equation modeling approach [90]. **Fig 1** schematically depicts the model applied in this study. Because our endogenous variables (*Trumpism*, *NoACC* and *NoVax*) are ordinal or dichotomous, we use a variation called generalized structural equation modeling (GSEM). By incorporating generalized linear modeling into the structural equation framework, GSEM permits multi-equation models with nonlinear link functions and non-Gaussian distribution families, such as ordered logistic regression (logistic link function, ordinal distribution family)—equivalent to binomial logit, if the dependent variables are dichotomies. Data management and calculation of GSEM models, with diagnostic tests and graphing of interaction effects, were carried out using Stata 16.1.

## 5. Results

### 5.1 GSEM modeling

Results from GSEM analysis modeling climate-change and vaccination rejection as functions of demographic and identity/belief characteristics, with Trump support as an intervening variable, appear in **Table 2**. GSEM applies generalized linear modeling (GLM) within a structural equation framework. In this case, the GLM aspect involves ordered logistic regression. Table 2 shows logit coefficients with their robust standard errors, the latter calculated by a generalized Huber/White/sandwich estimator. Stars summarize probabilities from *z* tests employing these standard errors.

**Table 2:** Logit coefficients (with robust standard errors), from GSEM using ordered logit regression. Estimation sample n = 1,061; variable definitions and coding are given in Table 1.

Endogenous variables			
Exogenous variables	*Trumpism*	*NoACC*	*NoVax*
*Age*	–0.022 (0.004)***	0.018 (0.004)***	–0.024 (0.006)***
*Female*	–0.226 (0.133)	0.091 (0.163)	0.556 (0.184)**
*White*	0.611 (0.169)***	–0.136 (0.204)	–0.068 (0.226)
*Income*	0.006 (0.062)	0.006 (0.070)	–0.214 (0.214)**
*Evangelical*	0.542 (0.160)***	0.207 (0.190)	0.029 (0.212)
*Education*	–0.111 (0.077)	–0.321 (0.094)***	–0.428 (0.113)***
*Ideology*	0.304 (0.047)***	0.213 (0.055)***	0.305 (0.062)***
*Education × ideology*	0.006 (0.035)	0.119 (0.042)**	0.111 (0.048)*
*Friends*	0.420 (0.125)***	–0.251 (0.168)	0.075 (0.180)
*Party*	0.990 (0.096)***	0.035 (0.128)	0.052 (0.150)
*Friends × party*	0.678 (0.134)***	0.576 (0.175)**	0.579 (0.193)**
*Conspiracist*	0.427 (0.064)***	0.060 (0.076)	0.239 (0.078)**
*Trumpism*		0.181 (0.042)***	0.096 (0.049)*

*p < 0.05

**p < 0.01

***p < 0.001 (two-tailed *z* tests).

As expected, conservatives, Republicans, whites and evangelicals are all significantly more likely to approve of Trump (hypothesis H1). Trump support also is more common among people receptive to seemingly unpolitical conspiracy beliefs—that NASA faked the Moon landings, or the Earth is really flat (H6). Finally, as detailed in section 5.2 on interactions, Trump support is higher among Republicans who say that most of their friends belong to the same party (H5).

Trump support in turn predicts both rejection of ACC and rejection of COVID-19 vaccination. Although the positive signs of these direct Trumpism effects are not surprising, their significance is noteworthy because the analysis already controls for other characteristics including ideology, party, race and religion, as well as *education×party* and *friends×party* interactions. Trumpism, as hypothesized, exhibits a direct effect on science rejection for both issues, net of the effects from other identity indicators (H2). Identification as white or evangelical, on the other hand, does not directly affect ACC or vaccine rejection—contrary to hypothesis H3.

Although Trumpism increases the odds of both climate and vaccine rejection (H1), its effects on climate are stronger. Several cross-cutting relationships may account for this difference. Vaccine rejection is greatest among younger respondents (opposite to climate rejection), presumably because older people have a sharper sense of their own mortality, are more accustomed to following medical advice, and face higher risks from COVID-19. Vaccine rejection also is greater among women. Finally, lower household income and openness to conspiracy beliefs increase the odds of rejecting vaccination, whereas neither income nor conspiracism directly affect climate views (supporting only the first part of H6).

Trumpism plays a significant role as intervening variable in our analysis, consistent with the conclusions of Shao and Hao [19]. This means that background factors might exert indirect (mediated) effects through Trumpism, in addition to whatever direct effects they have (H2a). Such indirect effects are approximated as products of coefficients along a sequence of paths from exogenous to final endogenous variables. Where the sequence includes one positive and one negative coefficient, the indirect effect is negative. Where the sequence includes either two positive or two negative coefficients, the indirect effect is positive. For ACC rejection, indirect effects through Trumpism significantly modify (strengthen or weaken) conclusions that might be drawn from the direct effects of most background characteristics (age, white, evangelical, ideology, party, same-party friends and conspiracist beliefs). For example, the total effect of evangelical identity on ACC rejection is significantly stronger if we consider not only its positive direct effect, but also its positive indirect effect through Trumpism: evangelicals are more likely to support Trump, and (even controlling for party, ideology, race etc.) Trump supporters are more likely to reject ACC. Both party and conspiricism show significant indirect effects through Trumpism on vaccine rejection as well. Indirect effects on vaccine rejection from age, identification as white or evangelical, ideology and same-party friends fall just short of significance (all 0.10 > *p* > 0.05).

**Fig 2:**
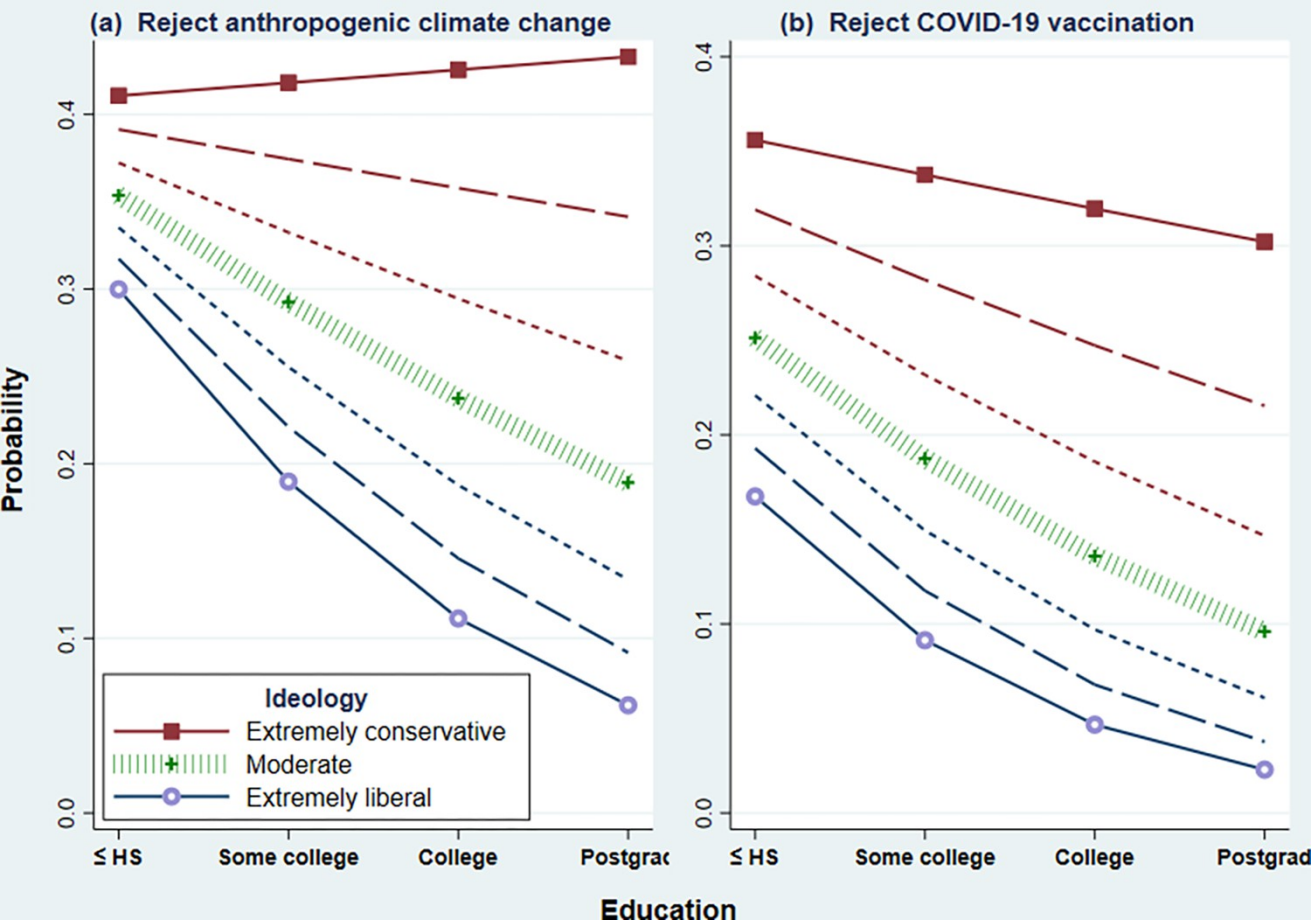
*Education×ideology* interaction effects on (a) probability respondent rejects reality of anthropogenic climate change, or (b) does not intend to get vaccinated for COVID-19. Marginal probabilities adjusted for other covariates, calculated from the GSEM model in Table 2.

Because the predictors *education*, *ideology*, *friends* and *party* appear also in interaction terms, their main effects in Table 2 can be interpreted as the effect of that variable when its interacting counterpart equals zero. For example, the main effects shown for *education* on *Trumpism*, *NoACC* and *NoVax* represent the effects of *education* when *ideology* = 0; that is, the effects of education among ideological moderates. Among moderates, education has little effect on Trump support. Even among moderates, however, education decreases the probability of rejecting ACC or vaccination—as visualized by the down-sloping “moderate” curves in **Fig 2A** and **2B**.

**Fig 3:**
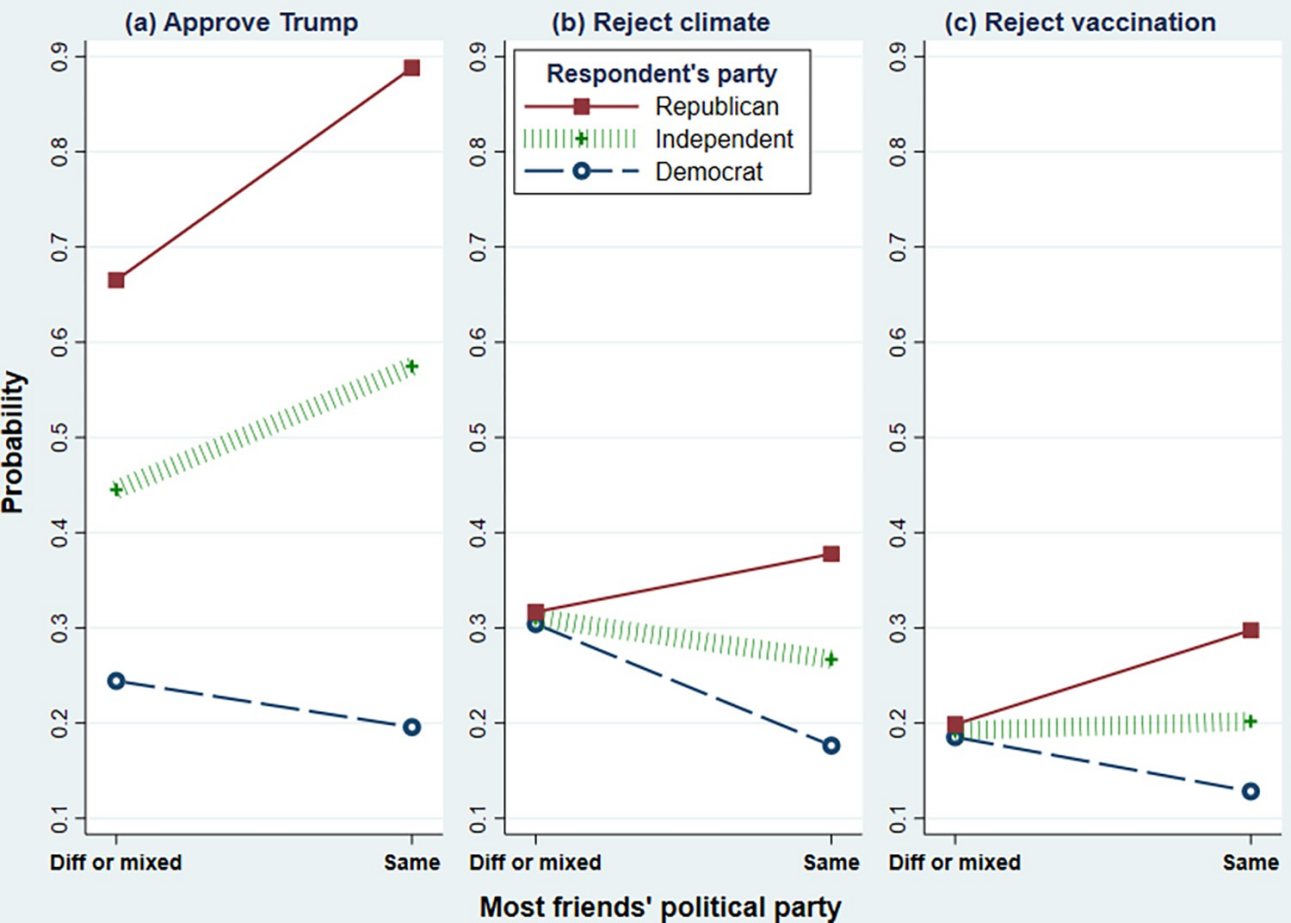
*Friends×party* interaction effects on probability respondent (a) strongly approves of ex-President Trump, (b) rejects reality of anthropogenic climate change, or (c) does not intend to get vaccinated for COVID-19. Marginal probabilities adjusted for other covariates, calculated from the GSEM model in Table 2.

Similarly, the main effects of having same-party friends correspond to the *friends* effect when *party* = 0; that is, the effect of same-party friends among independents. Having mostly same-party friends increases the probability that self-identified independents will approve of Trump (**Fig 3A**) but has little effect on their ACC or vaccination rejection (**Fig 3B** and **3C**).

### 5.2 Interaction effects

Views on climate change and vaccination are both predicted by *education×ideology* interactions in which education just slightly affects the probability of science rejection among extreme conservatives but lowers this probability among all other groups (H4). Fig 2 visualizes these two interactions as marginal probability plots, adjusted for other covariates in the GSEM model of Table 2. Fig 2A shows that, other things being equal, among extremely conservative respondents the probability of rejecting ACC rises slightly with education (from 0.41 to 0.43) but declines with education among all other groups. For example, the conditional probability of ACC rejection declines from 0.35 among moderates with high school education to 0.19 among moderates with college education. Fig 2B similarly shows that the conditional probability of rejecting COVID vaccination decreases slightly (from 0.36 to 0.30) with education among extreme conservatives but declines more steeply with education among all other groups (for example, from 0.25 to 0.10 among moderates).

Similar interactions have been seen in many studies of climate change and other environmental topics, so Fig 2A is not surprising. Such interactions involving COVID vaccine rejection (Fig 2B) have not previously been documented, however. A recent study by Shoots-Reinhard et al. [91] reports a complementary result: ideological differences in COVID-19 risk perceptions were most pronounced among individuals with higher verbal (but not numerical) ability. Individuals with greater verbal ability, like those with higher education, may be more responsive to partisan cueing.

Both climate-change and vaccination views exhibit greater partisan divisions among people with mostly same-party friends (*friends×party* interaction; hypothesis H5). Trumpism also is higher among people with mostly same-party friends. Fig 3 visualizes these statistically significant interactions. In each case, the gap between Republicans and Democrats widens when most of the respondent’s friends identify with the same party they do. Almost 90 percent of Republicans with mostly same-party friends approve of Trump, compared with fewer than 70 percent of Republicans with mixed or different-party friends (Fig 3A). Impacts on climate and vaccine rejection are smaller in absolute magnitude but have similar meaning. The Democrat–Republican gap in COVID vaccine or ACC rejection widens from about .01 (when they have mixed or different-party friends) to 0.17 or 0.20 (when they have mostly same-party friends).

### 5.3 Robustness of results

How stable are the findings described here? This section reviews diagnostic and goodness-of-fit statistics and evaluates some alternative model specifications.

By inflating standard errors (making estimates less precise), multicollinearity can raise problems in multivariate analyses. This is particularly a risk when interaction terms are involved. Testing for multicollinearity, we used ordinary least squares to approximate variance inflation factors (VIF) and tolerances (1/VIF) for independent variables in each equation of our GSEM analysis. Chatterjee and Hadi [92] write that VIF of 10.0 or higher indicate serious multicollinearity; other sources suggest that a VIF of 4.0 could be cause for concern. For the *NoACC* and *NoVax* equations in Table 2, VIF ranged from 1.04 to 2.85, with a mean of 1.61, well below thresholds for concern. VIF are even lower for the *Trumpism* equation: 1.02 to 2.44, with a mean of 1.51. These results indicate that despite the interaction terms, multicollinearity is not problematic for our analysis.

Goodness of fit is an ambiguous concept with logit and ordered-logit analyses, which lack a simple counterpart to linear regression’s explained-variance measure, R^2^. A variety of pseudo R^2^ statistics have been proposed, but these can give divergent results and should be interpreted alongside other goodness-of-fit statistics [93]. McFadden’s pseudo R^2^, the most widely used, equals 0.20 for the *NoACC* equation in Table 2. Correct classification rate for this NoACC equation is 77% with 87% specificity and 54% sensitivity. A Hosmer-Lemeshow goodness-of-fit χ^2^ test does not reject the fitted model, nor does a specification link test.

For the *NoVax* equation in Table 2, McFadden’s pseudo R^2^ equals 0.19; correct classification rate is 81% with 96% specificity and 23% sensitivity. As with *NoACC*, a Hosmer-Lemeshow goodness-of-fit χ^2^ test does not reject the fitted model, nor does a specification link test.

Unlike the binomial *NoACC* and *NoVax* equations, the equation for ordinal Trump support employs ordered logit regression, for which correct classification rates and Hosmer-Lemeshow tests are less appropriate. Pseudo R^2^ for this equation equals 0.21. Specification link tests suggest that we could obtain a better fit by using a dichotomous version of this variable (support Trump or not) instead of the 7-category ordinal measure employed. But a dichotomous indicator for Trumpism is substantively less appealing. It also works less well analytically: using dichotomous instead of ordinal Trump support worsens the fit of both *NoACC* and *NoVax* equations, as indicated by comparisons of their respective Bayesian information criteria (BIC). That is, ordinal Trump support predicts *NoACC* and *NoVax* better than dichotomous Trump support does. These results support keeping the ordinal version.

Two exogenous variables in our analysis, *education* (4 categories) and *party* (3 categories) were modeled as ordinal predictors having approximately linear effects. Such treatment follows many previous studies (for graphed examples involving climate change and vaccines, see Figs 2 and 3 in [53]) and yields parsimonious, interpretable results consistent with hypotheses. An alternative approach preferred by some authors would be to enter *education* and *party* as unordered categorical variables. Although using categorical *education* and *party* requires substantially more complicated models (six additional parameters in each equation), it does not improve fit. The categorical-predictor models yield almost the same pseudo R^2^ values and worse BIC.

## 6. Discussion

Trumpism’s impacts on two existentially consequential cases of science rejection, climate change and COVID vaccination, reinforce its importance as an emergent sociopolitical identity. Although Trump’s personal future is uncertain, his deep effects on US society are unlikely to go away soon; under some scenarios they could intensify. Even if support for Trump himself narrows, for example, elements of conspiracism and science rejection might become more pronounced among his core believers, or attach to new grievances and leaders. The potentially global consequences of science rejection by a leading US political party underline the need for continued scrutiny. An intervening-variable framework along the lines of Fig 1, and propositions supported by this analysis, could apply in studies of Trumpism beyond Trump.

Lower trust in scientists (generically) among US conservatives is well known [94–96], although sometimes coexisting with abstract expressions of trust in science itself. Surveys find liberal/conservative divisions across many specific fields besides climate and COVID—including disbelief in central propositions of biology, geology and astronomy; and lower trust in scientists regarding nuclear power, genetically modified organisms, environmental protection, renewable energy, forest management, and vaccination long before COVID [53,97–99]. Conservative (and Trump-supporter) distrust of epidemiologists emerged quickly during the 2016 Zika pandemic [32,100]. Bias across these major fields runs consistently in the same direction—contrary to early hypotheses that there might be an opposite bias among liberals against vaccines, nuclear power or genetically modified organisms. In contrast to most surveys, some experimental studies have reported items where liberals express greater skepticism—but that seems most common with counterfactual experimental prompts such as an invented scientific consensus that nuclear power is the most efficient form of alternative energy [101], or a fictional expert asserting that carrying concealed handguns decreases violent crime [102].

The breadth of real fields where science rejection occurs tends to undercut explanations based on scientific content in particular cases. Examples of content-based explanations include religious objections to science on evolution or the age of the Earth [103], or a liberal/conservative split on *impact science* vs. *production science* (e.g., science pointing out environmental or occupational health risks, vs. science that advances manufacturing or commerce) [104–106]. Disproportionately conservative science resistance across many fields also argues against explanations based on real conduct by scientists themselves, who in most areas are little known to the public.

Hamilton and Safford [18] considered alternative hypotheses as they might apply to one specific case, the rapid fall of trust in the US Centers for Disease Control (CDC) observed among Republicans during early months of the COVID-19 pandemic. They concluded that only elite cues—specifically, changing pronouncements from Trump—could explain the abruptness and scale of partisan decline. Other studies have found significant Trump-supporter effects regarding climate, COVID and other topics [14,19,31–33]. Trumpism exacerbates the broader trend toward conservative science rejection across many fields.

Matt Motta and coauthors recently studied “canine vaccine hesitancy” (CVH), a rising distrust among dog owners regarding long-established vaccines against rabies [107]. Their 2023 survey of 2,200 US adults found surprisingly high levels of doubts and non-vaccination, with concerns borrowed from misinformation about human vaccines. For example, 37 percent believed that vaccination might cause pets to develop autism. Political dimensions of this phenomenon were not the original paper’s focus, but their dataset, also published, includes a question on 2020 voting. CVH is almost twice as common among Trump voters compared with Biden voters (60 vs. 33 percent). In multivariate analysis (by the author of this paper, not Motta et al.), Trump vs. Biden voting dominates other background factors (political party, gender, age, education, religion and race) as the strongest predictor of CVH. This example illustrates the ease with which politicized rejection in one area of science can generalize or spill over to other areas. It also highlights Trumpism as a key element in this process, extending to topics where Trump himself has said nothing.

Although conservative distrust of science does not apply to every field, it is demonstrably widespread and fits with broad trends toward anti-intellectualism [108] or rejection of expertise [109], populist impulses that can be mobilized to support economic or political goals [110]. The scientific method itself, a system for critically testing ideas against reality, will seem objectionable when it fails to support identity-bound convictions. In their study of online discourse among flat Earthers, Diaz Ruiz and Nilsson [111] note the importance of grievances and group identification, which make fact-checking irrelevant. Similar observations could apply more widely, including conspiratorial claims about climate and COVID. In the case of climate change and COVID, preexisting biases against scientists were reinforced by messaging from economic and political elites serving interests such as fossil fuel use or Trump’s re-election.

Trump approval correlates with other conspiracy or science-rejecting views. Trumpists indicate higher agreement not only with flat Earth and Moon landing conspiracies, but also that vaccinations implant tracking microchips, and COVID-19 dangers have been exaggerated by scientists. At the same time, they express lower agreement with scientific conclusions that the Earth is billions of years old, humans evolved from earlier forms of life, human activities are changing the climate, or vaccines are mostly beneficial [41].

Trump’s expressed positions have sometimes changed rapidly, including a reversal on COVID-19 vaccinations—from his administration’s initiative (Operation Warp Speed, intended to spur vaccine development) to post-administration opposition, when it seemed President Biden could get credit. Trump’s views on the Centers for Disease Control reversed even faster, in just a few months [18]. In both cases, Trump’s followers echoed his reversals. To some degree, the content of Trumpism hinges on whatever Trump says. Among supporters surveyed for a CBS News poll in August 2023, 71 percent reported feeling that what Trump tells them is true—much higher than the fractions expressing trust in their friends and family, conservative media, or religious leaders [112]. Noting that such attitudes insulate Trump supporters even from other conservative viewpoints, one observer wrote that “Trumpworld is a bubble within a bubble” [113]; another remarked that “If reality contradicts Trump, reality is wrong” [114]. Science and expertise remain outside the control of this bubble, and often in opposition to it. Unfortunately, there are deadly consequences to having a large fraction of the US public reject expertise regarding anthropogenic climate change, pandemics and other hard realities.

As with any study, the findings here depend on the data at hand—a US survey conducted in mid-2021. There is much scope for cross-national comparisons. Outside the US, climate change and COVID are salient issues with ideological divisions [115–117], although the reality of these problems may be less disputed by mainstream parties. US left/right party or ideological divisions do not necessarily map onto the politics of other countries. Trumpism might, however, have analogues elsewhere in populist leaders or movements that combine grievances and anti-immigrant sentiments with authoritarianism and rejection of mainstream liberal democratic values [118,119].

The 2021 date of our US data also is an important limitation. The phenomena studied here—rejection of climate change, COVID-19 vaccination, and admiration for former president Trump—are potentially subject to change. Indeed, temporal caveats apply to any public-opinion research, and perhaps more so now than in the past. The stability of conclusions from earlier surveys must be an empirical question. On the other hand, similarities shown here between the social bases of ACC and COVID vaccination rejection, one older and one comparatively recent phenomenon, suggest a degree of stability that could apply to future science-linked issues as well.

## 7. Conclusions

GSEM analysis of a nationally representative US survey conducted in mid-2021 finds both commonalities and differences in the social bases of rejecting anthropogenic climate change and rejecting COVID-19 vaccination. Prominent similarities include:

$ Conservative ideology and Trumpism directly increase the likelihood of science rejection in both cases.
$ Identification as white or evangelical do not directly affect science rejection, but do so indirectly through Trumpism.
$ Science rejection on both issues becomes less common with higher education. However, the effects of education are significantly stronger among moderates and liberals.
$ Having mostly same-party friends increases the probability of science rejection among Republicans.

Notable contrasts between ACC and COVID-19 vaccine rejection include:

$ The probability of rejecting ACC increases with age, whereas the probability of vaccine rejection decreases with age.
$ Women are more likely than men to reject vaccination, but the genders are roughly similar (other things being equal) with regard to climate change.
$ Vaccine rejection occurs more often in low-income households. Income has no impact on climate-change views.
$ Receptivity to conspiracy beliefs directly increases the likelihood of vaccine rejection, while having mainly an indirect effect (through Trumpism) on climate rejection.

Indirect effects through Trumpism on both ACC rejection and COVID-19 vaccine rejection add to the direct effects of white and evangelical identity, ideology, party, conspiracism, and the friends×party interaction—strengthening the positive total effects in each case. These conclusions are robust within our 2021 survey dataset but invite comparisons with other issues, other countries, and new data, as political alignments, media reporting, and public perceptions on these issues evolve.

## Supporting information

S1 File Stata-format POLES 2021 survey dataset. (DTA)

S2 File Do-file for GSEM analyses. (DO)
